# Phylogenetic Relatedness Determines Heterospecific Pollen–Pistil Compatibility and Reproductive Outcome in the Apocarpous Species *Sagittaria trifolia* (Alismataceae)

**DOI:** 10.1002/ece3.72866

**Published:** 2026-01-05

**Authors:** Si‐Yan Zou, Sen‐Tao Lyu, Ting Yu, Tian‐Yuan Zhang, Xin Zan, Xiao‐Fan Wang

**Affiliations:** ^1^ Laboratory of Plant Systematics and Evolutionary Biology, College of Life Sciences Wuhan University Wuhan China; ^2^ Biomedical Research Institute Hubei University of Medicine Shiyan China; ^3^ State Key Laboratory of Plant Diversity and Specialty Crops, Institute of Botany Chinese Academy of Sciences Beijing China

**Keywords:** extragynoecial compitum, heterospecific pollen deposition, phylogenetic distance, pollen–pistil interaction, reproductive interference, *Sagittaria*

## Abstract

Pollinator sharing among coflowering plants can reduce plant fitness through heterospecific pollen (HP) deposition. Compatible HP is expected to cause stronger reproductive interference, but the factors determining HP–pistil compatibility and its reproductive consequences remain poorly understood. Moreover, in some apocarpous taxa, the extragynoecial compitum, a basal pore formed by the incomplete fusion of carpel margins, can enhance reproductive assurance by facilitating conspecific pollen (CP) tube reallocation across free pistils. What remains unclear is how these taxa balance the trade‐off between this benefit and the potential risk of reproductive interference from intercarpellary heterospecific pollen (HP) tube growth. In this study, widely distributed apocarpous *Sagittaria trifolia* with typical extragynoecial compitum was selected as a pollen recipient. Hand‐pollination experiments were performed with 42 distributed and coflowering HP donors to assess pollen–pistil interaction and HP effects on seed quantity. Results showed that HP–pistil compatibility was independent of species origin (native/alien), pollen size, and aperture number of the HP donor. Instead, it was negatively correlated with phylogenetic distance: closely related HP exhibited higher compatibility with the pistil and caused greater reductions in seed set. Nevertheless, CP consistently sired over 78% of seeds whether applied simultaneously with or after HP. In the half‐and‐half pollination treatments with CP and compatible HP, CP sired significantly more than 50% of seeds. These indicated that CP advantage and intercarpellary CP tube growth collectively mitigate interspecific pollen interference. However, these compensatory mechanisms were less effective when faced with alien congeners. Our results provide a good basis for understanding the variation in HP‐mediated fitness costs and shed light on how apocarpous lineages with extragynoecial compitum adaptively tolerate HP interference and maintain reproductive success.

## Graphical abstract

This study investigates heterospecific pollen (HP) effects in *Sagittaria trifolia* , an apocarpous plant with an extragynoecial compitum. HP–pistil compatibility and resulting seed set reduction were greatest from phylogenetically close donors, not influenced by origin or pollen traits. Despite this, conspecific pollen (CP) consistently outcompeted even compatible HP for ovules due to pollen advantage and intercarpellary growth, siring over 78% of seeds when mixed or applied after HP, demonstrating mechanisms mitigating reproductive interference.

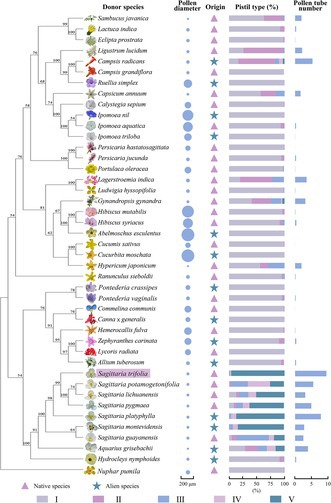

## 1 Introduction

Sympatric coflowering plants often share pollinators, and consequently heterospecific pollen (HP) deposition is common (Morales and Traveset 2008; Moreira‐Hernández and Muchhala 2019). The surface of stigma cells serves as the receptive site of pollen grains, playing a key role in discriminating between conspecific pollen (CP) and HP (Hülskamp et al. 1995; Edlund et al. 2004). Stigma–pollen recognition reactions determine pollen–pistil compatibility, enabling the rejection of pollen from inappropriate species while allowing the adhesion, hydration, and germination of compatible pollen, as well as subsequent pollen tube penetration through the stigma, style, and ultimately into the ovules (Lan et al. 2023). Compatible HP tends to impose greater fitness costs on recipient plants via direct interference with CP at three key sites within the pistil: the stigma, style, and ovule. At the stigma, germinated HP can induce the mechanical closure of touch‐sensitive stigmas (Waser and Fugate 1986; Zou et al. 2022) or physically block the stigmatic surfaces (Galen and Gregory 1989), thereby inhibiting the adhesion and germination of CP. Within the style, HP tube growth may cause physical clogging or exert allelopathic effects that suppress CP tube elongation (Murphy 2000; Brown and Mitchell 2001). At the ovule level, the compatible HP tubes, particularly those from phylogenetically closely related species, can successfully achieve fertilization, leading to ovule usurpation or hybridization (Burgess et al. 2007; Zou et al. 2023). Such reproductive interference, due to the absence of interspecific incompatibility against HP, may cause population decline and competitive exclusion of vulnerable species in natural populations (Nishida et al. 2014). Exploring the key factors involved in HP–pistil compatibility can help to better understand the variation in fitness consequences of HP receipt.

Interspecific pollen–pistil interaction may depend on phylogenetic relatedness between pollen donor and recipient. Closely related species tend to have similar stigma morphology and pollen‐stigma recognition systems, resulting in the misidentification of HP as CP (Lan et al. 2023). Thus, in contrast to phylogenetically distant related HP, closely related HP is more likely to achieve pollen adhesion, germination, pollen tube growth, and ovule penetration (Martin 1970; Zou et al. 2022). It can be predicted that both HP–pistil compatibility and HP effects on reproductive fitness will increase with decreasing phylogenetic distance, particularly when assessed across multiple HP donors spanning a continuous phylogenetic gradient.

Beyond the evolutionary history, the coexistence history is also critical in mediating HP–pistil compatibility. Long‐term sympatric coflowering species can develop enhanced tolerance to detrimental HP effects through prolonged post‐pollination interaction via pollinator sharing and HP receipt (Arceo‐Gómez et al. 2015). By contrast, newly sympatric species lacking coexistence history are predicted to exhibit novel pollen–pistil interactions. On the one hand, geographical isolation is sufficient to maintain species boundaries between allopatric plant species (Tang et al. 2022), potentially resulting in a weaker pollen–pistil barrier. Thus, exotic HP often causes more severe reproductive interference than native HP (Arceo‐Gómez and Ashman 2016). On the other hand, long‐term geographical isolation may promote genetic differentiation and species diversification (Wen and Jansen 1995), potentially strengthening pollen–pistil barriers between native and exotic species. Therefore, it remains to be tested whether HP–pistil compatibility and the resulting HP effects are related to the species origin of the HP donor.

Furthermore, certain morphological pollen traits, particularly pollen size and aperture number, may serve as critical determinants of pollen–pistil compatibility interactions (Ashman and Arceo‐Gómez 2013; Zou et al. 2022). Pollen size of HP influences the matching and adhesion between HP and stigmatic papillae of the recipient plant, thereby affecting pollen–pistil interactions (McLernon et al. 1996; Zou et al. 2022). Smaller‐grained HP exhibits enhanced adhesion capacity between stigma papillae, facilitating efficient absorption of water and nutrients required for successful germination, while larger‐grained HP becomes mechanically impeded by the papillary architecture, resulting in germination failure (Zou et al. 2022). Compared with larger‐grained HP, smaller‐grained HP may require fewer nutrients for initiating germination. Furthermore, since apertures serve as the channels for the transfer of water and nutrients as well as pollen tube emergence in the process of pollen germination (Punt et al. 2007), pollen grains with more apertures exhibit a greater likelihood of hydration and germination than pollen grains with fewer apertures (Dajoz et al. 1991, 1993). Thus, HP–pistil compatibility and the effects of HP receipt on reproductive success are predicted to increase with smaller pollen size but decrease with a greater number of pollen apertures.

**FIGURE 1:**
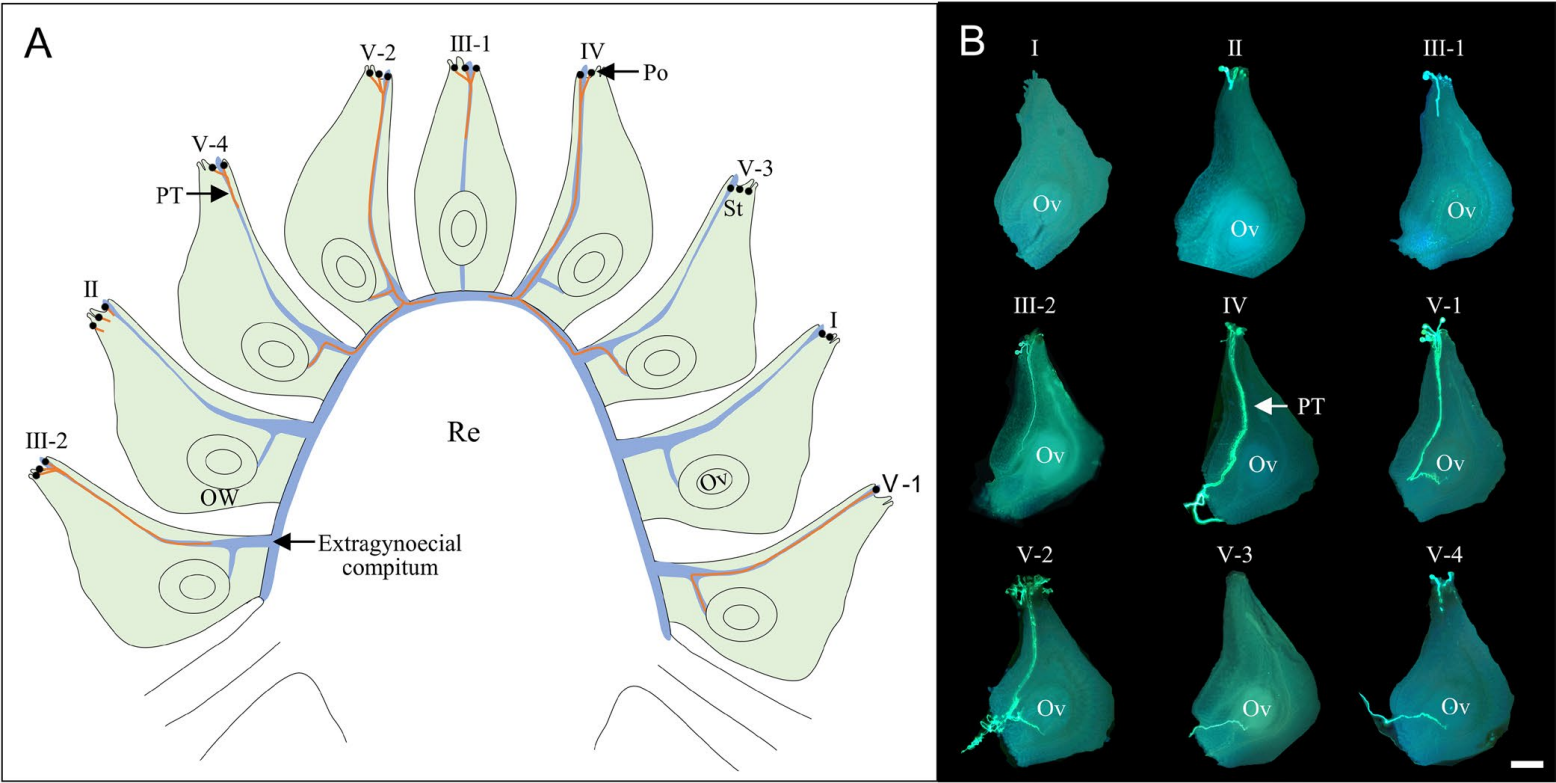
Intercarpellary pollen tube growth and categories of pistil based on pollen germination and tube development in *Sagittaria trifolia* . (A) Longitudinal section through a female flower of *S. trifolia* , showing intercarpellary pollen tube growth within the gynoecium via the extragynoecial compitum. (B) Fluorescence micrographs showing different categories of pistil: (I) pistil with ungerminated pollen grains on the stigma; (II) pistil with pollen grains beginning to germinate on the stigma; (III‐1, III‐2) pistils with pollen tubes arriving at different parts of the style; (IV) pistil with pollen tubes not growing toward the ovule but growing directly into the receptacle through the extragynoecial compitum; (V‐1, V‐2, V‐3, V‐4) pistils with pollen tubes reaching the ovule. All pistils are oriented with the stigma above and the ovule below. Ov, ovules; OW, ovary wall; Po, pollen grains; PT, pollen tube; Re, receptacle; St, stigma. Scale bar, 200 μm.

In addition to the four potential factors mentioned above, interspecific pollen–pistil interactions and the resulting HP effects might also vary depending on pistil types (syncarpous vs. apocarpous). In syncarpous pistils, pollen tube growth is limited within a single pistil due to the complete closure of the ovary base. By contrast, intercarpellary pollen tube growth is common in some apocarpous species, such as *Artabotrys* (Annonaceae; Chen et al. 2025), *Illicium*, *Kadsura*, and *Schisandra* (Schisandraceae; Williams et al. 1993; Lyew et al. 2007; Du and Wang 2012), *Ranalisma* and *Sagittaria* (Alismataceae; Wang et al. 2002, 2006, 2012), as well as *Sedum* (Crassulaceae; Wang et al. 2012). In these taxa, pollen tubes from one pistil can grow between the free pistils within the gynoecium through the extragynoecial compitum, which refers to a basal opening formed by the incomplete fusion of carpel margins (Figure 1; Huang et al. 2014). Such pollen tube reallocation allows CP tubes to exit a fully fertilized pistil and reach the unfertilized ovules in other pistils, thereby promoting fruit and seed production (Wang et al. 2012; Huang and Wang 2017). However, the existence of extragynoecial compitum may confer potential HP compatibility, amplifying the risk of reproductive interference by increasing the opportunities for HP tubes (especially near phylogenetically related HP tubes) to occupy more ovules (Lyu et al. 2017). What remains unclear is how apocarpous plants with extragynoecial compitum balance the trade‐off between the benefit of reproductive assurance and the risk of reproductive interference.

*Sagittaria* (Alismataceae) represents a classic basal angiosperm lineage that preserves the ancestral and typical apocarpous gynoecium morphology (Chen 1989). With approximately 40 species worldwide (Ito et al. 2020), *Sagittaria* includes seven native and three invasive species in China. In this study, the widely distributed native species *S. trifolia* was selected as a pollen recipient owing to its high floral abundance and natural exposure to HP deposition in both wild and artificial communities (Fei et al. 2022; Zou et al. 2023; Zou Si‐Yan, unpublished data). In this study, hand‐pollination experiments were performed with 42 sympatrically distributed and coflowering HP donors to assess pollen–pistil interaction and HP effects on seed production. We included phylogenetic distance, species origin (native or alien), and pollen trait as explanatory variables. In particular, we aimed to address the following questions: (1) Does pistil compatibility differ in response to different HP donors? (2) How do phylogenetic distance, species origin, pollen size, and pollen aperture number mediate HP–pistil compatibility? (3) How do different HP donors affect seed set? Our objectives were to determine the variation in fitness costs of HP receipt in apocarpous plant species and to understand the ecological and evolutionary consequences of plant–plant post‐pollination interactions.

## 2 Materials and Methods

### 2.1 Study System

#### 2.1.1 HP Recipient Species

*Sagittaria trifolia* (Alismataceae) is an aquatic perennial herb, widely distributed in marshes, wetlands, and rice fields in the south of China, and typically flowers from May to October (Chen 1989). It is monoecious and protogynous, bearing both female and male flowers (Figure S1), with female flowers grown in the proximal whorls of the paniculate or racemose and bloom first, while male flowers are in the distal whorls and bloom subsequently (Chen 1989; Huang et al. 2006). The bowl‐shaped flowers consist of three green sepals and three white petals, blooming in the morning and only opening for a single day (Chen 1989). The female flowers contain numerous apocarpous pistils clustered on the globose receptacle (Figure S1), and each pistil has a single ovule (Chen 1989). The achene has a beak formed by the persistent style, and its seeds appear horseshoe‐shaped with brown testa (Chen 1989). Pollination of *S. trifolia* primarily relies on generalist pollinators, including bees and flies (Huang et al. 2006). Additionally, *S. trifolia* can propagate vegetatively through corms and stolons, enabling rapid population expansion (Chen 1989).

#### 2.1.2 HP Donor Species

In the communities in Guchi (27°50′ N, 118°07′ E, Wuyishan, Fujian, China), Wuhan University (30°32′ N, 114°21′ E, Wuhan, Hubei, China), and Wuhan Botanical Garden (30°32′ N, 114°25′ E, Wuhan, Hubei, China), *S. trifolia* experiences interspecific pollen transfer with co‐flowering and co‐occurring plant species via pollinator sharing (Zou Si‐Yan, unpublished data). To enhance the generalizability of the results, a broader range of HP donors from diverse families, which varied in pollen traits and geographic origins, were included in the experimental design. A total of 42 sympatric species were selected as HP donors (Table S1), comprising six congeneric species (within *Sagittaria* spp.), two confamilial species from different genera (Alismataceae, non‐*Sagittaria*), and 34 species from phylogenetically distant related families (non‐Alismataceae). The native/exotic status of all 42 species was verified through the Flora of China (https://www.iplant.cn/), as documented in Table S1. Preliminary experiments confirmed that among all HP donors, only *S. platyphylla* could produce fully developed hybrid seeds with *S. trifolia* , whereas all other crosses resulted in seed abortion (Zou et al. 2023; Zou Si‐Yan, unpublished data). Thus, we could attribute any well‐developed seeds produced in the mixed‐pollination experiments to CP.

#### 2.1.3 Study Sites

In late May 2023, about 60 seedlings of *S. trifolia* were randomly collected from a monospecific population in the rice field of the Hybrid Rice Cultivation Base and transplanted to the Garden of Greenhouse in Wuhan University. The selected individuals were size‐standardized to eliminate the potential size‐dependent effects on experimental outcomes. Ten individuals from each of 10 donor species that were not established in Wuhan University were also transplanted into the experimental cultivation area. Sampling information was detailed in Table S1. All other donor species were naturally or artificially established within Wuhan University, thus requiring no transplantation. For cultivated plant individuals, normal water and fertilizer management were carried out regularly, with light and temperature provided naturally. All fieldwork was conducted from June to August in 2023.

### 2.2 Evaluation of HP–Pistil Compatibility

The conspecific and 42 heterospecific pollen donors were selected to assess pollen–pistil compatibility. Female flowers of *S. trifolia* and pollen donor flower buds were previously bagged with mesh netting to exclude pollinators at dusk 1 day before pollination. Pollinations were performed on sunny days between 8:00 and 10:00 to coincide with high pollen viability and stigma receptivity. Since pollination tools like brushes can damage the stigma and significantly reduce fruiting probability and seed number in *S. trifolia* (Xie et al. 2021), this study employed direct contact pollination. Anthers from different donors were collected in advance and placed in a petri dish. Then, female flowers of *S. trifolia* were pollinated by directly applying either CP or HP to the stigmas of the entire gynoecium using freshly dehisced anthers in saturating amounts. Each day of the experiment, all newly opened female flowers of *S. trifolia* were randomly assigned to intraspecific or interspecific crosses. Pollinated flowers were bagged again to avoid subsequent visitation by pollinators. At 8 h after pollination, each female flower was harvested and fixed in FAA solution (70% (v/v) alcohol: acetic acid: formalin, 90:5:5). Preliminary studies indicated that an 8 h duration was sufficient for complete pollen germination and pollen tube growth in *Sagittaria* species (Lyu et al. 2017; Zou et al. 2023). To minimize the influence of individual variations on the results, the female flowers of *S. trifolia* in each cross were distributed across more than three maternal individuals, and pollen grains were collected from over five donor individuals.

The collected gynoecium was softened with 20% NaOH for 5 h and stained with 0.1% aniline blue for 1 h, using a procedure modified from Zou et al. (2023). Pollen germination and pollen tube growth were then observed under a fluorescence microscope (Olympus BX43; Olympus Co., Tokyo, Japan). During storage and solution treatment, some pollen grains may detach from the stigma. These grains were collected by centrifugation and transferred to a slide for examination. Microscopic analysis showed that nearly all detached pollen grains failed to germinate. For each cross, 10 female flowers (approximately 60 pistils per flower) were randomly observed. For each flower, 20 pistils were randomly selected from the observed pistils to count the number of pollen tubes in the style. In total, 430 flowers and 26,810 pistils were examined for all crosses.

Based on pollen germination and pollen tube growth status, pistils were categorized into different types (Figure 1; Table S2). Pollen tube growth in each pistil was evaluated on a compatibility score (Jewell et al. 2012): 0 = unsuccessful germination; 1 = pollen tube growth halting in the stigma; 2 = pollen tubes extending to the style or receptacle; 3 = pollen tubes reaching the ovule. To evaluate HP–pistil compatibility at different stages of post‐pollination progression, five indices were used: HP–stigma compatibility, HP–style compatibility, HP–ovule compatibility, number of pollen tubes per pistil, and overall HP–pistil compatibility. Stigma, style, and ovule compatibility to HP were calculated as the percentage of pistils with pollen tubes that had reached the stigma, style (or beyond), and ovule, respectively. For example, the presence of pollen tubes in the style indicates successful compatibility with both the stigma and style, with similar logic applying to subsequent stages. The overall compatibility index was computed as the weighted sum of compatibility scores across all observed pistils for each species pair.

### 2.3 Interspecific Pollination and Estimation of Seed Set

Four compatible HP donors, representing contrasting phylogenetic distance and origin, were selected based on their frequent occurrence on *S. trifolia* stigmas in the field: *S. potamogetonifolia* (native congener), *S. montevidensis* (exotic congener), *Lagerstroemia indica* (native distant relative), and *Campsis radicans* (exotic distant relative). This experimental design allows examination of the effects of both phylogenetic distance (congeners vs. distant relatives) and species origin (native vs. exotic) on pollination outcomes.

Artificial pollination was conducted during the peak flowering period. Based on the natural pollen load of 7.05 ± 0.49 (mean ± SE) grains per single pistil observed in the field (Zou Si‐Yan, unpublished data), a saturated amount of pollen was applied in all treatments to ensure full coverage of the stigmatic surface. A total of 26 hand‐pollination treatments were performed as follows: (1) pollination with CP (the whole gynoecium was pollinated with adequate CP), (2) pollination with half CP (one half of the gynoecium was pollinated with adequate CP and the other half remained unpollinated), (3–22) delayed CP pollination (the gynoecium was pollinated first with HP, and then with adequate CP after 0, 0.5, 1, and 2 h), (23–26) half‐and‐half pollination with CP and HP (one half of the gynoecium was pollinated with adequate CP and the other half with adequate HP; Figure S1C). Pollination procedures followed the protocol described in the previous section, and the flowers were bagged after pollination. A total of 520 female flowers (20 flowers per pollination combination) were used in the experiment.

Approximately 20 days post‐pollination, mature aggregate fruits were harvested and dried for 5 h in an electric heating constant temperature drying box (GZX‐9030 MBE, Boxun Co., Shanghai, China) at 50°C. The achenes of each fruit were dissected under a stereoscope (Olympus SZX16, Olympus Co., Tokyo, Japan) and classified into three categories based on achene size and embryonic development (Huang 2003): aborted, partially developed, and fully developed. Only fully developed achenes were counted as successful seed formation, while partially developed and aborted achenes were both considered unsuccessful. Since one pistil produced only one ovule in *Sagittaria* (Chen 1989), seed formation was equal to achene formation. The seed set was calculated as the ratio between the number of fully developed achenes and the total number of achenes (Table S3). A total of 228,450 achenes were examined across all pollination treatments.

### 2.4 Calculation of Phylogenetic Distance

One nuclear gene (ITS) and two chloroplast genes (*mat*K and *rbc*L) were selected to calculate the pairwise phylogenetic distance between *S. trifolia* and 42 HP donors. All sequences were downloaded from the NCBI database, with all GenBank accession numbers detailed in Table S1. When sequences for a species were unavailable in GenBank, its synonym (previous taxonomic name) was used for searching. Because five sequences of four species remained unobtainable even using synonyms, a representative from the genus was used. Given the extensive phylogenetic span among the studied species contained in this study, the use of congeneric sequences was unlikely to influence the results (Zheng et al. 2018). Multiple sequence alignment was performed using the ClustalW algorithm in MEGA 11.0, with poorly aligned terminal regions trimmed (Tamura et al. 2021). The three DNA regions were concatenated into a supermatrix using PhyloSuite (Zhang et al. 2020). Based on this supermatrix, a neighbor‐joining tree was then constructed in MEGA 11.0 using the Kimura two‐parameter model, with branch support assessed through 500 bootstrap replicates (Tamura et al. 2021). *Nuphar pumila* (Nymphaeaceae) was used to root the tree. Finally, pairwise phylogenetic distances between *S. trifolia* and HP donors were calculated using the Kimura two‐parameter model in MEGA 11.0 (Tamura et al. 2021) and are expressed in units of substitutions per site.

### 2.5 Measurement of Pollen Size and Pollen Aperture Number

Pollen grains from all species were separately collected and preserved in 1.5 mL microcentrifuge tubes containing 1 mL FAA solution. Following multiple centrifugations, pollen grains were washed with distilled water several times, treated with 10% NaOH solution for 1 h, and stained with 0.1% aniline blue for 1 h. Pollen morphology was observed, and pollen aperture number was counted under a fluorescence microscope (Olympus BX43; Olympus Co., Tokyo, Japan). High‐resolution images were captured, and polar axis lengths (A) and equatorial axis lengths (B) of the pollen grains were then measured for 20 randomly selected grains per species using ImageJ FIJI 2.0 (National Institutes of Health, Bethesda, Maryland, USA). Pollen volume was calculated using the ellipsoid volume formula *V* = 4/3πAB^2^. For species lacking distinct apertures under fluorescence microscopy, supplementary observations were conducted using scanning electron microscopy (S‐3400 *N* + PP3000T; Quorum Technologies, Tokyo, Japan) following the protocols detailed in Zou et al. (2022). To characterize directional differences in floral traits between donor–recipient pairs, trait distances were computed by subtracting recipient ( *S. trifolia* ) values from donor values for both pollen size and aperture number. Negative trait distances indicate donor values smaller than those of the recipient, vice versa.

### 2.6 Statistical Analysis

Shapiro–Wilk and Levene's tests were used to assess the normality of data distribution and homogeneity of variances, respectively. The significant differences in pollen tube growth among different recipient–donor species combinations were determined using Kruskal–Wallis *H* tests, and significant differences in the post hoc multiple comparison were identified with Dunn's post hoc test.

To exclude the influence of phylogenetic relationship, the phylogenetic signals of pollen size and pollen aperture number were tested with the R package phytools (Revell 2012), using Pagel's lambda as a metric of phylogenetic independence. The absence of significant phylogenetic signal (*p* > 0.05) permitted exclusion of phylogenetic corrections in subsequent analyses. Three types of generalized linear mixed models (GLMMs) were implemented to analyze the effects of four factors (phylogenetic distance, species origin, pollen size, and aperture number) on five pollen–pistil compatibility indicators, with donor species as a random effect. For HP–stigma/style/ovule compatibility, a GLMM with a binomial distribution and a logit link function was performed, using the proportion of specific pistil type (i.e., number of specific pistil type/total observed pistils) as the response variable. For the number of HP tubes per pistil, a Poisson GLMM was conducted with a log link function. As for overall HP–pistil compatibility, a GLMM was performed with a normal distribution and an identity link function, in which the compatibility score was included as the response variable.

Additionally, the differences in seed set among conspecific and delayed CP pollination treatments were identified using Mann–Whitney *U* tests for pairwise comparison and Kruskal–Wallis *H* tests adapted with Dunn's post hoc test in the post hoc multiple comparison. One‐way ANOVA adapted with the Games–Howell post hoc test was used to detect seed set differences among conspecific, half CP, and half‐and‐half pollination treatments with CP and HP. In half‐and‐half pollination, *t*‐tests were used to examine the deviations of the exact seed set from the expected proportion based on the proportion of CP‐pollinated pistils on all pistils (50%).

All data analyses were conducted using SPSS version 28.0 (IBM, Armonk, New York, USA) and R version 4.4.1 (R Development Core Team, University of Auckland), with a *p* value < 0.05 considered statistically significant.

## 3 Results

### 3.1 Pollen–Pistil Compatibility Between HP and *S. trifolia* Pistils

**FIGURE 2:**
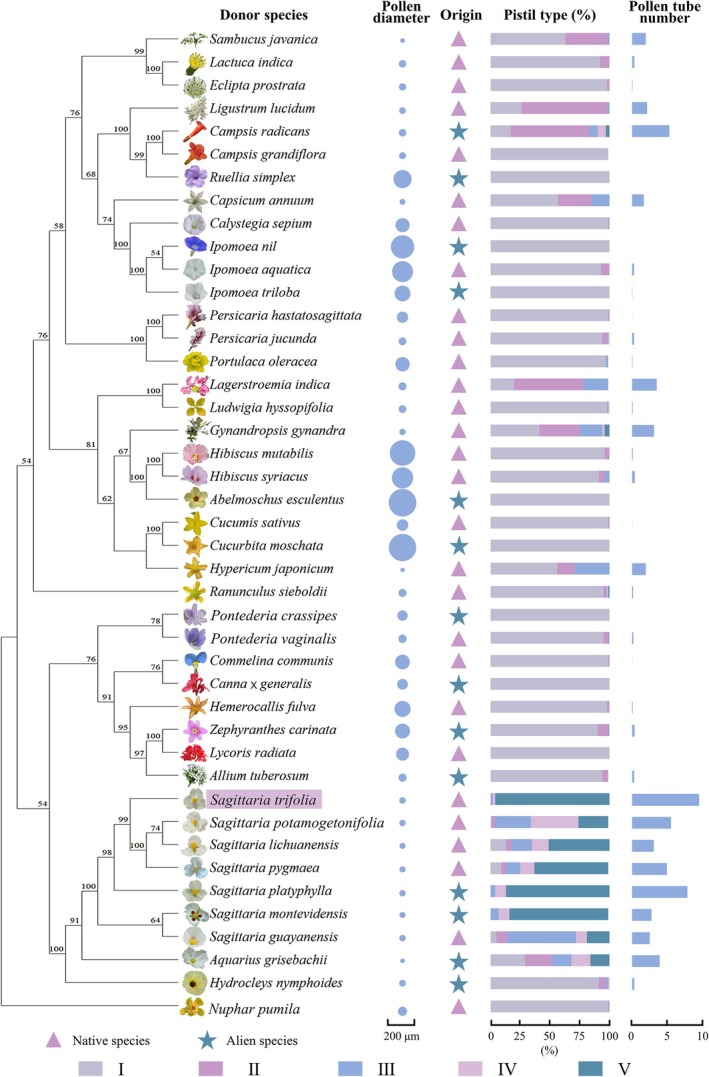
Phylogenetic distribution of heterospecific pollen germination and pollen tube growth on the gynoecium of *Sagittaria trifolia* . The phylogenetic tree depicts 42 donor species used in this study, with pollen diameter represented by circle size (average of polar and equatorial axes) and species origin indicated by symbols (native: Purple triangles; alien: Green stars). Bars represent the proportion of pistils: (I) pistil with ungerminated pollen grains; (II) pistil with pollen grains beginning to germinate; (III) pistils with pollen tubes arriving at style; (IV) pistil with pollen tubes not growing toward the ovule but growing directly into the receptacle through the extragynoecial compitum; (V) pistils with pollen tubes reaching the ovule. Pollen tube number, the mean number of pollen tubes supported by a single pistil of *S. trifolia* .

**FIGURE 3:**
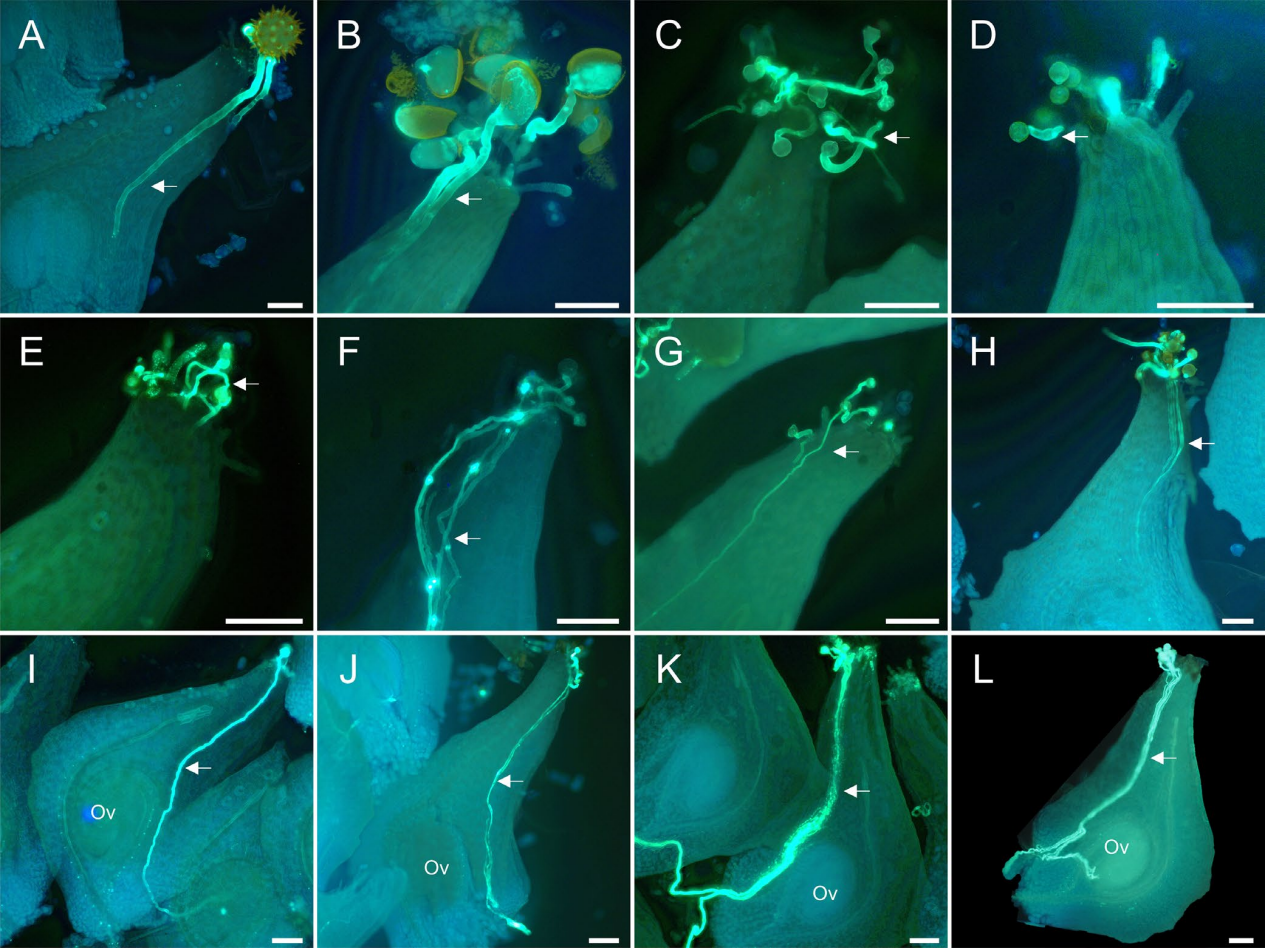
Pollen germination and pollen tube growth of heterospecific pollens in the pistils of *Sagittaria trifolia* under a fluorescence microscope. Pollen grains from (A) *Hibiscus syriacus* (Malvaceae), (B) *Zephyranthes candida* (Amaryllidaceae), (C) *Capsicum annuum* (Solanaceae), (D) *Sambucus javanica* (Viburnaceae), (E) *Ligustrum lucidum* (Oleaceae), (F) *Gynandropsis gynandra* (Cleomaceae), (G) *Hypericum japonicum* (Hypericaceae), (H) *Lagerstroemia indica* (Lythraceae), (I) *Campsis radicans* (Bignoniaceae), (J) *Aquarius grisebachii* (Alismataceae), (K) *S. potamogetonifolia* (Alismataceae), and (L) *S. montevidensis* (Alismataceae). All pistils are oriented with the stigma above and the ovule below. The white arrows indicate heterospecific pollen tubes. Ov, ovules. Scale bar, 100 μm.

Overall, the compatibility between different HP and *S. trifolia* pistils varied considerably. Among the 36 phylogenetically distant related HP donors from different genera (non‐*Sagittaria*), most failed to germinate on *S. trifolia* stigmas (Figure 2). Only eight distantly related HP donors showed substantial germination success on *S. trifolia* stigma, including *Aquarius grisebachii* (Alismataceae), *Campsis radicans* (Bignoniaceae), *Capsicum annuum* (Solanaceae), *Gynandropsis gynandra* (Cleomaceae), *Hypericum japonicum* (Hypericaceae), *Lagerstroemia indica* (Lythraceae), *Ligustrum lucidum* (Oleaceae), and *Sambucus javanica* (Viburnaceae). *Aquarius grisebachii*, from the same family as *S. trifolia* , showed the highest compatibility, with its pollen tubes reaching the ovules (Figure 2). In contrast, all other compatible distantly related HP from different families could only grow as far as the stigma or style (Figure 3). Furthermore, some pollen tubes from *C. radicans* and *A. grisebachii* exhibited a reallocation phenomenon within the *S. trifolia* gynoecium, where they passed through the extragynoecial compitum and entered the receptacle (Figure 3). Additionally, certain HP tubes showed aberrant morphology or growth paths (Figure S2).

Closely related HP from the same genus displayed significantly higher compatibility with *S. trifolia* pistils than phylogenetically distant HP did (Figure 2). Their pollen grains germinated abundantly, and tubes grew into styles and ovules. Similar to CP, congeneric HP all displayed the pollen tube reallocation phenomenon. Although compatible, the number of HP tubes supported by a single pistil was significantly lower than that of CP tubes (*H* = 24.113, *p* < 0.001). Among congeneric donors, alien HP exhibited higher compatibility with *S. trifolia* pistils than native HP (Figure 2). On average, 86.95% and 83.37% of *S. trifolia* pistils permitted pollen tubes from the non‐native species *S. platyphylla* and *S. montevidensis* to reach the ovules, showing no significant difference compared with the proportion receiving CP tubes. Conversely, significantly fewer ovules received pollen tubes from native congeneric HP than from CP (*H* = 53.753, *p* < 0.001). Moreover, a considerable portion of pollen tubes from native congeneric donors could only grow as far as the style or receptacle of *S. trifolia* , particularly those from *S. guayanensis* subsp. *lappula* and *S. potamogetonifolia* (Figure 2).

### 3.2 Effects of Phylogenetic Distance, Species Origin, Pollen Size, and Pollen Aperture Number on HP–Pistil Compatibility

Pollen size and pollen aperture number of HP donors comprised in the present study exhibited substantial variation (Table S1). The results of GLMMs showed that phylogenetic distance was significantly and negatively related to five pollen–pistil compatibility indicators (Table 1). By contrast, species origin, pollen size, and pollen aperture number were not associated with any pollen–pistil compatibility indicators.

**TABLE 1:** Summary of the generalized linear mixed models that assessed the effects of four factors on five indicators of heterospecific pollen–pistil compatibility in the gynoecium of *Sagittaria trifolia* .

Indicator of HP–pistil compatibility		Phylogenetic distance	Pollen size	Pollen aperture number	Species origin (native or alien)
HP–stigma compatibility	Estimate	−21.963	0	0.001	−0.757
	SE	3.812	0	0.015	0.855
	*z*	−5.76	−1.835	0.090	−0.885
	*p*	**< 0.001**	0.067	0.928	0.376
HP–style compatibility	Estimate	−25.773	0	−0.011	−0.014
	SE	3.988	0	0.022	0.928
	*z*	−6.463	−1.586	−0.507	−0.015
	*p*	**< 0.001**	0.114	0.612	0.988
HP–ovule compatibility	Estimate	−21.496	0	0.154	0.750
	SE	5.936	0	0.134	1.038
	*z*	−3.622	−1.470	1.150	0.723
	*p*	**< 0.001**	0.142	0.251	0.470
Number of PT per pistil	Estimate	−11.183	0	0.001	−0.892
	SE	2.950	0	0.012	0.669
	*z*	−3.791	−1.961	0.065	−1.333
	*p*	**< 0.001**	0.051	0.948	0.183
HP–pistil compatibility scores	Estimate	−6.994	0	0	−0.052
	SE	0.695	0	0.003	0.155
	*t*	−10.063	−1.168	0.016	−0.334
	*p*	**< 0.001**	0.243	0.987	0.738

*Note:* Phylogenetic distance, the phylogenetic distance between *S. trifolia* and HP donors. Pollen size, the pollen volume of HP. Pollen aperture number, the aperture number of HP. Species origin, the species origin (native or alien) of the HP donor. Significant differences (*p* < 0.05) are shown in bold type.

Abbreviations: HP, heterospecific pollen; PT, pollen tube.

### 3.3 Seed Set in Conspecific and Delayed Conspecific Pollination Treatments

**FIGURE 4:**
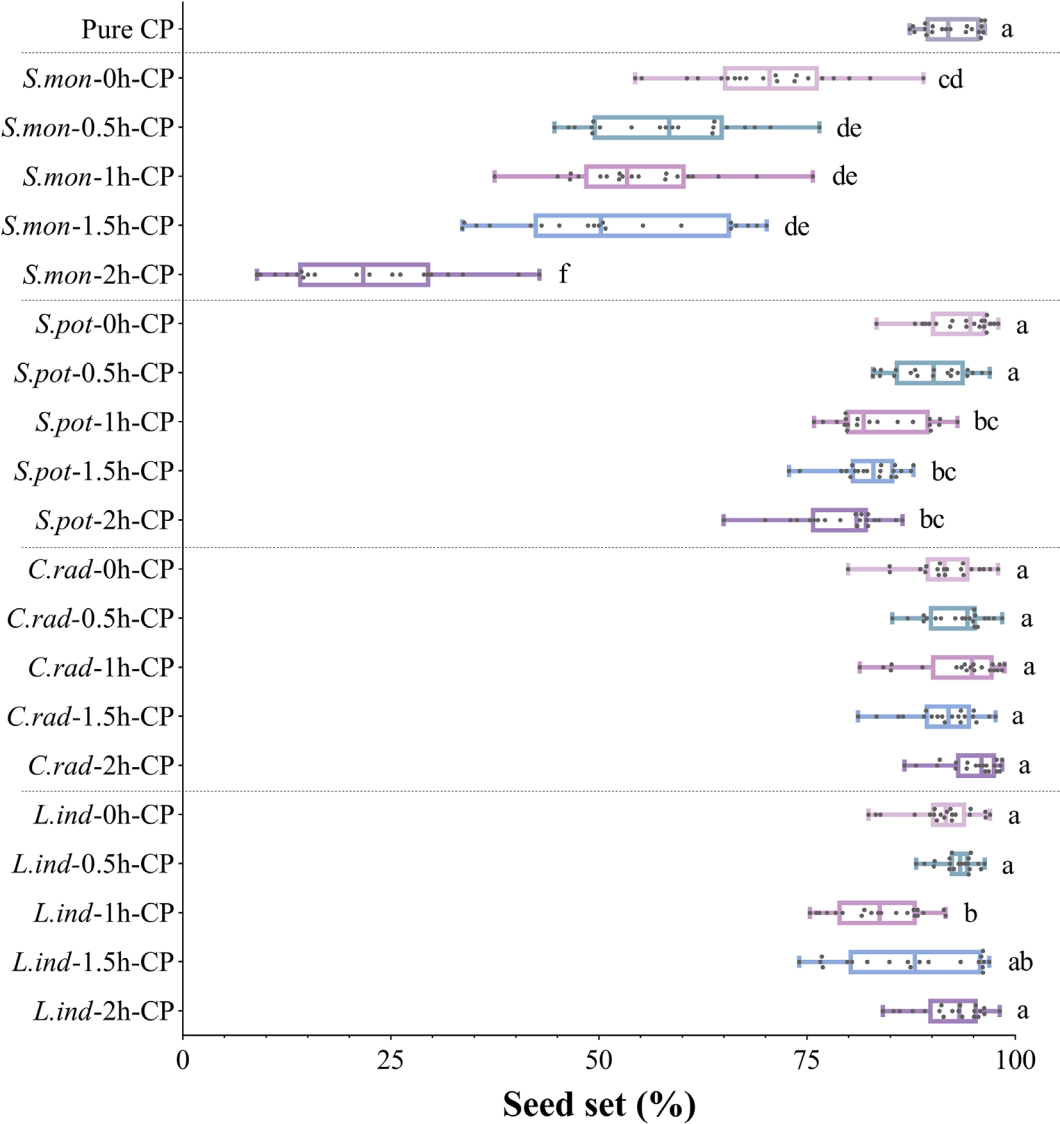
Seed set of *Sagittaria trifolia* under conspecific and delayed conspecific pollination treatments. Abbreviations: *S. mon*, *S. montevidensis* ; *S. pot*, *S. potamogetonifolia*; *C. rad*, *Campsis radicans* ; *L. ind*, *Lagerstroemia indica* . Pure CP, the whole gynoecium was pollinated with adequate conspecific pollen (CP). For delayed conspecific pollination, the gynoecium was first pollinated with adequate heterospecific pollen (HP), followed by adequate CP after 0, 0.5, 1, and 2 h. For example, the *S. mon*‐1 h‐CP treatment indicates that HP from *S. montevidensis* was applied first, followed by CP application 1 h later. Values with the same lowercase letters are not significantly different (*p* > 0.05).

Significant differences in seed set of *S. trifolia* were observed among different HP donors in CP delayed pollination treatments (*H* = 259.796, *p* = 0.016, Figure 4). Overall, two phylogenetically distant related HP types ( *C. radicans* and *L. indica* ) did not exert significant negative effects on the seed set of *S. trifolia* , with mean seed set remaining consistently high (> 87%) regardless of whether their pollen was applied simultaneously with CP or 0.5–2 h prior. The sole exception was the *L. indica* ‐1 h‐CP treatment, which showed a slightly lower seed set (83.69% ± 5.31%) compared to conspecific pollination (*Z* = −4.608, *p* < 0.001), potentially due to operational errors during pollination.

Two congeneric HP ( *S. montevidensis* and *S. potamogetonifolia*) significantly reduced the seed set of *S. trifolia* (Figure 4). Notably, pollen from the exotic congener *S. montevidensis* caused the most severe reproductive interference, with all CP delayed pollination (0–2 h time intervals) treatments showing significantly lower seed set compared to conspecific pollination. Seed set decreased significantly from 70.23% ± 8.62% in *S. montevidensis‐*CP simultaneous pollination to 22.35% ± 10.02% when CP application was delayed by 2 h. By contrast, pollen grains from native congener *S. potamogetonifolia* caused moderate reproductive interference. Seed set showed no significant reduction at 0 and 0.5 h time interval between *S. potamogetonifolia* pollen grains and CP application compared to controls, while it progressively decreased with longer delays. Nevertheless, seed set still achieved 78.79% ± 5.31% even when CP application was delayed by 2 h.

### 3.4 Seed Set in Half‐And‐Half Pollination Treatments With CP and HP

**FIGURE 5:**
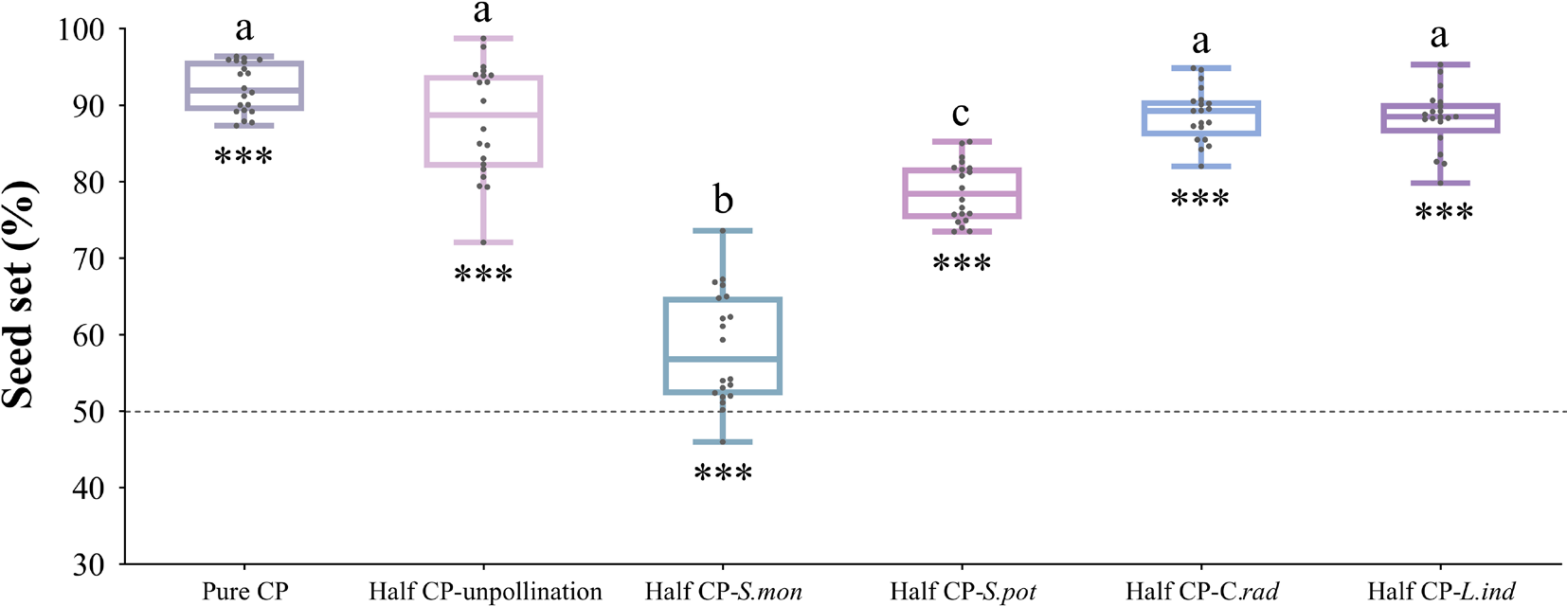
Seed set of *Sagittaria trifolia* under half‐and‐half pollination treatments with conspecific and heterospecific pollens. *C. rad*, *Campsis radicans* ; CP, conspecific pollen; *L. ind*, *Lagerstroemia indica* ; *S. mon*, *S. montevidensis* ; *S. pot*, *S. potamogetonifolia*. Pure CP, the whole gynoecium was pollinated with adequate CP. Half CP‐unpollination, one half of the gynoecium was pollinated with adequate CP, and the other half remained unpollinated. Half CP‐HP, one half of the gynoecium was pollinated with adequate CP, and the other half with adequate heterospecific pollens. For example, the half CP‐*S. mon* treatment indicates that one half of the gynoecium was pollinated with adequate CP and the other half with adequate pollen grains from *S. montevidensis* . Asterisks show that the seed set is significantly different from 50% (dashed line) (***p < 0.001). Different letters indicate significant differences among treatments (*p* < 0.05).

Seed set under half‐and‐half pollination treatments varied significantly (*F*_5,114_ = 116.722, *p* < 0.001, Figure 5). Three split‐pollination treatments with CP and either *C. radicans* , *L. indica* , or remaining unpollinated achieved equivalent seed set to conspecific pollination, indicating CP tubes can navigate through the extragynoecial compitum to fertilize ovules in the opposite half of the gynoecium. In contrast, the two congeneric HP donors, *S. montevidensis* and *S. potamogetonifolia*, significantly decreased seed set to 58.35% and 78.74%, yet these values still remained significantly above the 50% expected value (*t* = 5.015, *p* < 0.001; *t* = 10.907, *p* < 0.001).

## 4 Discussion

Reproductive success of most plants relies on generalist pollinators, so a high degree of pollinator sharing and HP transfer is common among coflowering species within plant communities (Morales and Traveset 2008; Moreira‐Hernández and Muchhala 2019). Evaluating the costs and benefits of pollinator sharing for coflowering plants, as well as the underlying factors that determine HP effects, is essential to understanding the mechanisms of species coexistence and community assembly (Ashman and Arceo‐Gómez 2013). In this study, we found that HP–pistil compatibility was mediated by phylogenetic distance between pollen donor and recipient, rather than species origin, pollen size, and pollen aperture number of HP donor. Compared to phylogenetically distant HP, closely related HP exhibited significantly higher compatibility with *S. trifolia* pistils, consequently causing more substantial reductions in seed set. However, the CP advantage and intercarpellary pollen tube growth provided more opportunities for CP tubes to outcompete HP and occupy ovules, thereby minimizing the potential reproductive interference. To the best of our knowledge, this is the first attempt to explore the pistil compatibility to HP and the underlying factors in apocarpous species.

Since Ashman and Arceo‐Gómez (2013) first proposed that phylogenetic relatedness and floral traits might influence pollen–pistil compatibility and thereby mediate HP effects on recipient reproductive success, subsequent studies have demonstrated that HP effects correlate more strongly with specific floral traits (e.g., pollen size, stigma size, pollen–ovule ratios, and style length) than with phylogenetic distance (Lanuza et al. 2021; Malecore et al. 2021; Zou et al. 2022). However, our results indicated that phylogenetic distance was a significant predictor of HP–pistil compatibility. Specifically, we confirmed that evolutionarily closely related donors displayed more detrimental effects on seed production than distantly related ones, a pattern consistent with two earlier studies (Arceo‐Gómez and Ashman 2016; Streher et al. 2020). Moreover, our findings diverge from those reported by Zou et al. (2022), who found that pollen size and aperture number affected the germination success of HP in *Campsis radicans* (Bignoniaceae). This discrepancy may be attributed to the differences in experimental scope, as our study incorporated a broader taxonomic range of HP donors. Nevertheless, it is important to acknowledge that our conclusions are based on controlled laboratory conditions, which may not fully capture the complexity of pollen–pistil interactions under natural environments. To better understand the evolutionary consequences of pollinator sharing and HP transfer, future studies should extensively evaluate the factors regulating HP–pistil compatibility and HP effects on reproductive success in natural communities, using a wider range of donor–recipient species pairs.

Although HP–pistil compatibility was independent of species origin when averaged across all donors, alien congeners exhibited significantly higher compatibility with *S. trifolia* pistils than native congeners, achieving greater ovule penetration and causing more severe reductions in seed production. These results suggested that *Sagittaria* species may have evolved specific mechanisms suppressing HP tube growth from native congeneric donors, thereby maintaining reproductive isolation. This pattern aligns with coevolutionary theory, which posits that long‐term sympatric species can develop enhanced HP tolerance through pollinator‐mediated selection (Arceo‐Gómez et al. 2015). Conversely, for native‐alien species combinations lacking a shared coevolutionary history, stigmas likely face novel biochemical recognition challenges, which may account for the observed higher HP–pistil compatibility. Therefore, alien species are more harmful donors than natives (Arceo‐Gómez and Ashman 2016). The invasive alien *S. platyphylla* causes severe reproductive interference in native species via hybridization and genetic introgression (Zou et al. 2023), while *S. montevidensis* does so through ovule discounting. Such high compatibility of alien HP with native pistils and its consequent reduction of reproductive success can be an effective mechanism facilitating alien plant invasion and success within native coflowering communities. The molecular basis of these differential compatibility patterns between the alien and native congeners requires elucidation.

Our results indicated that the pistils of apocarpous *S. trifolia* exhibited phylogenetically constrained compatibility, showing higher acceptance of congeneric HP, similar to the syncarpous model *Arabidopsis thaliana* where intergeneric HP fails to penetrate stigmas (Lan et al. 2023). However, a key divergence was observed after pollen adhesion: unlike in *A. thaliana* , *S. trifolia* pistils permitted pollen germination and even stylar penetration by eight intergeneric HP species. Remarkably, pollen tubes from some phylogenetically distant HP that reached the style were able to enter the receptacle via the extragynoecial compitum, mirroring the growth pattern of CP and congeneric HP. The higher compatibility in *S. trifolia* is likely facilitated by the extragynoecial compitum. In contrast to the restricted pollen tube growth within a closed pistil in syncarpous taxa, this structure provides a passage for pollen tubes to exit the pistil and grow freely across the receptacle to other pistils (Williams et al. 1993; Wang et al. 2002, 2006, 2012; Lyew et al. 2007; Du and Wang 2012; Chen et al. 2025). These pronounced differences suggest deep evolutionary divergence in pollen‐stigma recognition systems and ovule‐directed pollen tube guidance mechanisms between syncarpous and apocarpous taxa, warranting comparative studies.

Pollen–pistil interaction can function as a form of gametic isolation to limit HP effects on female fitness, including stigma–HP incompatibility and suppression of pollen tube growth for HP (Moreira‐Hernández and Muchhala 2019). Stigmas of angiosperms enable selective rejection of mismatched HP, typically phylogenetically distant related HP (Hülskamp et al. 1995; Edlund et al. 2004). In apocarpous *S. trifolia* , the stigma can recognize and filter most distantly related HP by impairing adhesion and preventing water and nutrient uptake, thereby inhibiting germination and forming the first physiological barrier. This reduction in physical clogging risk ensures successful CP germination even when CP arrives after HP. At the style level, the upper portion of the style further filters HP tubes, restricting their access to the lower style. This mechanism can reduce the likelihood of stylar clogging and serves as an effective strategy to mitigate interspecific reproductive interference. At the ovule level, three distinct mechanisms function to limit interspecific reproductive interference. First, ovules may secrete nonspecific chemical signals that repel HP tubes from native congeners within the same pistil, redirecting them via the extragynoecial compitum and receptacle tissue toward ovules of adjacent pistils. This extended growth path diminishes the opportunity for HP to occupy ovules, a phenomenon also observed in native S*agittaria* species combinations, including *S. trifolia* and *S. pygmaea* (Lyu et al. 2017; Fei et al. 2022). Second, CP outcompetes HP in pollen tube growth and seed siring, likely due to faster pollen tube elongation of CP tubes or higher attrition rates of HP tubes (Montgomery et al. 2010). Such CP advantage is common in angiosperms and involves a series of intricate molecular and cellular processes (Takeuchi and Higashiyama 2012; Zhong et al. 2019). Third, CP tubes can utilize extragynoecial pathways to access adjacent pistils and fertilize ovules that would otherwise be fertilized or aborted by HP, thereby securing reproductive assurance. These results support the view that intercarpellary pollen tube growth mediated by the extragynoecial compitum in apocarpous angiosperms provides reproductive assurance under unpredictable pollination conditions (Huang 2003; Wang et al. 2006, 2012; Huang and Wang 2017; Fei et al. 2022). However, when confronted with highly compatible HP from alien congeners, the effectiveness of CP precedence and intercarpellary pollen tube growth in minimizing reproductive interference appears relatively limited. Our results demonstrate that apocarpous lineages can evolve compensatory adaptive mechanisms to offset fitness costs from HP receipt. Given that apocarpous taxa with extragynoecial compitum occur across multiple branches of angiosperm phylogeny (Wang et al. 2012), future studies should validate these findings in a broader range of lineages.

In conclusion, our study demonstrated that HP–pistil incompatibility and HP effects on reproductive success in apocarpous *S. trifolia* were phylogenetically constrained. In contrast, species origin, pollen size, and pollen aperture number did not exert impacts on pollen–pistil interaction. Pistil incompatibility to HP, combined with CP advantage and intercarpellary CP tube growth, provided CP tubes with greater opportunities to occupy ovules. These results suggested that while the apocarpous pistil maintains relatively open pollen tube pathways via the extragynoecial compitum and receptacle tissue, it also reinforces tolerance to HP to mitigate potential interspecific reproductive interference. The reliance of *S. trifolia* on generalist pollinators results in long‐term exposure to HP, which appears to have selected for the evolution of post‐pollination tolerance mechanisms that offset the associated fitness costs. However, when confronted with recently introduced alien congeners, with which no shared evolutionary history exists, the absence of such adaptive mechanisms results in severe reproductive interference. This study improves our understanding of the variation in fitness costs from HP deposition and how apocarpous lineages with extragynoecial compitum are adaptive to intense interspecific pollen competition. Future research should elucidate the mechanisms of interspecific pollen–pistil interactions in more apocarpous taxa to understand how pollinator sharing influences pollen–pistil recognition and floral diversification.

## Author Contributions

**Si‐Yan Zou:** conceptualization (equal), formal analysis (equal), investigation (equal), methodology (equal), visualization (equal), writing – original draft (equal), writing – review and editing (equal). **Sen‐Tao Lyu:** conceptualization (equal), formal analysis (equal), investigation (equal), methodology (equal), visualization (equal), writing – original draft (equal), writing – review and editing (equal). **Ting Yu:** conceptualization (supporting), investigation (equal), writing – original draft (supporting). **Tian‐Yuan Zhang:** conceptualization (supporting), investigation (supporting), writing – original draft (supporting). **Xin Zan:** conceptualization (supporting), investigation (supporting), writing – original draft (supporting). **Xiao‐Fan Wang:** conceptualization (lead), funding acquisition (lead), methodology (equal), project administration (lead), supervision (lead).

## Funding

This work was supported by the National Natural Science Foundation of China, 31970250 and Faculty Development Grants from Hubei University of Medicine, 2024QDJZR013.

## Conflicts of Interest

The authors declare no conflicts of interest.

## Supporting information


**Figures S1–S2:** ece372866‐sup‐0001‐FiguresS1‐S2.docx.

**Tables S1–S3:** ece372866‐sup‐0002‐TablesS1‐S3.xlsx.

## Data Availability

All data are provided as Supporting Information.
