# Supplementary material for: Phylogenetic Relatedness Determines Heterospecific Pollen–Pistil Compatibility and Reproductive Outcome in the Apocarpous Species Sagittaria trifolia (Alismataceae)
Source: Ecol Evol. 2026 Jan 5;16(1):e72866. doi: 10.1002/ece3.72866 (PMC12771588; doi:10.1002/ece3.72866)

**Figure S1:** Plant individual and floral morphology of *Sagittaria trifolia*. (A) Plant individual, (B) male flower, and (C) female flower. The white dashed line indicates the division for the half-and-half pollination treatments.


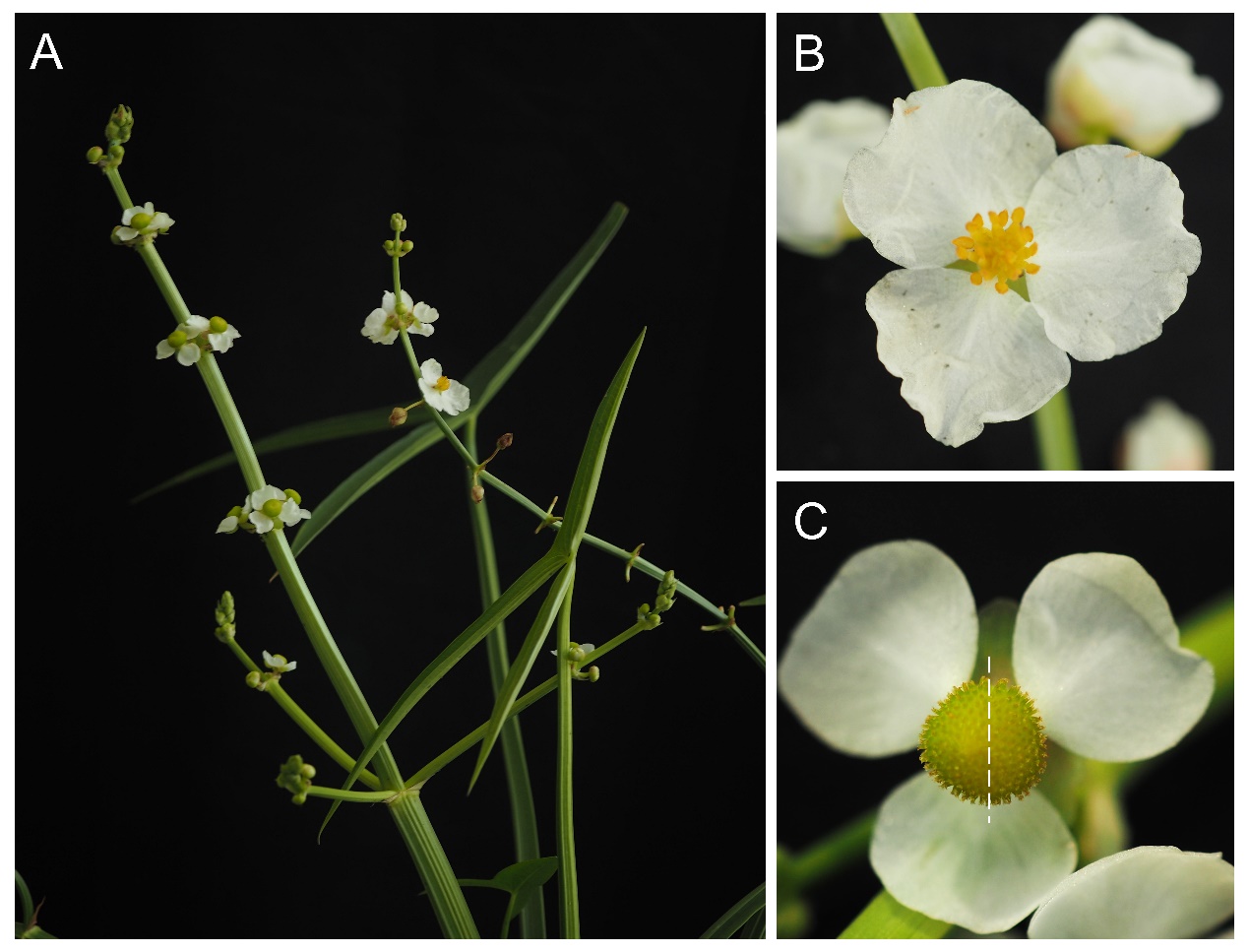


**Figure S2:** Incongruity phenomenon of heterospecific pollen tube growth in the pistils of *Sagittaria trifolia* under a fluorescence microscope. Some pollen tubes of *S. platyphylla* (A) and *Aquarius grisebachii* (B) became twisted ("rope-like") or displayed tortuous growth near the ovary before ultimately entering *S. trifolia* ovules. Pollen tubes from *A. grisebachii* (C-E) and *Campsis radicans* (F) frequently looped within the lower stylar canal, and then either returned to the upper stylar region or entered the receptacle through the extragynoecial compitum. Remarkably, some *C. radicans* pollen tubes that regrew from receptacle tissue into adjacent pistils followed incorrect trajectories — instead of turning toward ovules, they grew directly into the upper stylar canal (F). All pistils are oriented with the stigma above and the ovule below. The white arrows indicate incongruity form or path of heterospecific pollen tubes. Ov, Ovules. Scale bar, 100 μm.


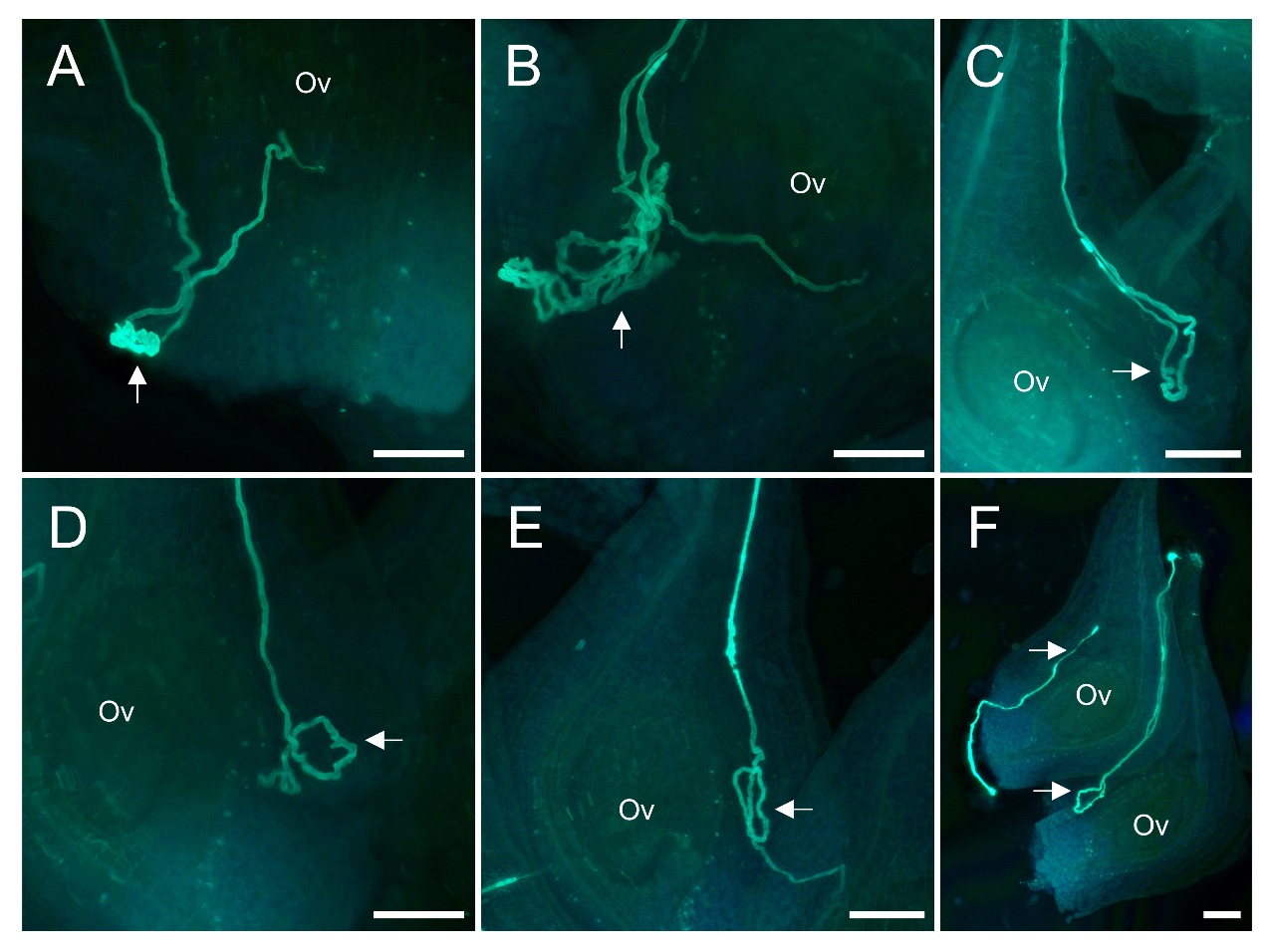

Supplement: Supplementary file 1 — Figures S1–S2: ece372866‐sup‐0001‐FiguresS1‐S2.docx. [file ECE3-16-e72866-s001.docx]
